# The approaches to measuring the potential spatial access to urban health services revisited: distance types and aggregation-error issues

**DOI:** 10.1186/s12942-017-0105-9

**Published:** 2017-08-23

**Authors:** Philippe Apparicio, Jérémy Gelb, Anne-Sophie Dubé, Simon Kingham, Lise Gauvin, Éric Robitaille

**Affiliations:** 1 Centre Urbanisation Culture Société, Institut National de la Recherche Scientifique, 385 Sherbrooke Street East, Montréal, QC H2X 1E3 Canada; 2 Department of Social and Preventive Medicine, Faculty of Medicine, University of Montréal, P.O. Box 6128, Downtown Station, Montréal, QC H3C 3J7 Canada; 3 GeoHealth Laboratory, Department of Geography, University of Canterbury, Private Bag 4800, Christchurch, 8140 New Zealand; 4 Institut National de Santé Publique du Québec, 190 Boulevard Crémazie Est, Montréal, QC H2P 1E2 Canada

**Keywords:** Accessibility of health services, GIS, Sensitivity analysis, Uncertainty analysis, Cartesian distance, Network distances

## Abstract

**Background:**

The potential spatial access to urban health services is an important issue in health geography, spatial epidemiology and public health. Computing geographical accessibility measures for residential areas (e.g. census tracts) depends on a type of distance, a method of aggregation, and a measure of accessibility. The aim of this paper is to compare discrepancies in results for the geographical accessibility of health services computed using six distance types (Euclidean and Manhattan distances; shortest network time on foot, by bicycle, by public transit, and by car), four aggregation methods, and fourteen accessibility measures.

**Methods:**

To explore variations in results according to the six types of distance and the aggregation methods, correlation analyses are performed. To measure how the assessment of potential spatial access varies according to three parameters (type of distance, aggregation method, and accessibility measure), sensitivity analysis (SA) and uncertainty analysis (UA) are conducted.

**Results:**

First, independently of the type of distance used except for shortest network time by public transit, the results are globally similar (correlation >0.90). However, important local variations in correlation between Cartesian and the four shortest network time distances are observed, notably in suburban areas where Cartesian distances are less precise. Second, the choice of the aggregation method is also important: compared with the most accurate aggregation method, accessibility measures computed from census tract centroids, though not inaccurate, yield important measurement errors for 10% of census tracts. Third, the SA results show that the evaluation of potential geographic access may vary a great deal depending on the accessibility measure and, to a lesser degree, the type of distance and aggregation method. Fourth, the UA results clearly indicate areas of strong uncertainty in suburban areas, whereas central neighbourhoods show lower levels of uncertainty.

**Conclusion:**

In order to accurately assess potential geographic access to health services in urban areas, it is particularly important to choose a precise type of distance and aggregation method. Then, depending on the research objectives, the choices of the type of network distance (according to the mode of transportation) and of a number of accessibility measures should be carefully considered and adequately justified.

## Background

**Fig. 1:**
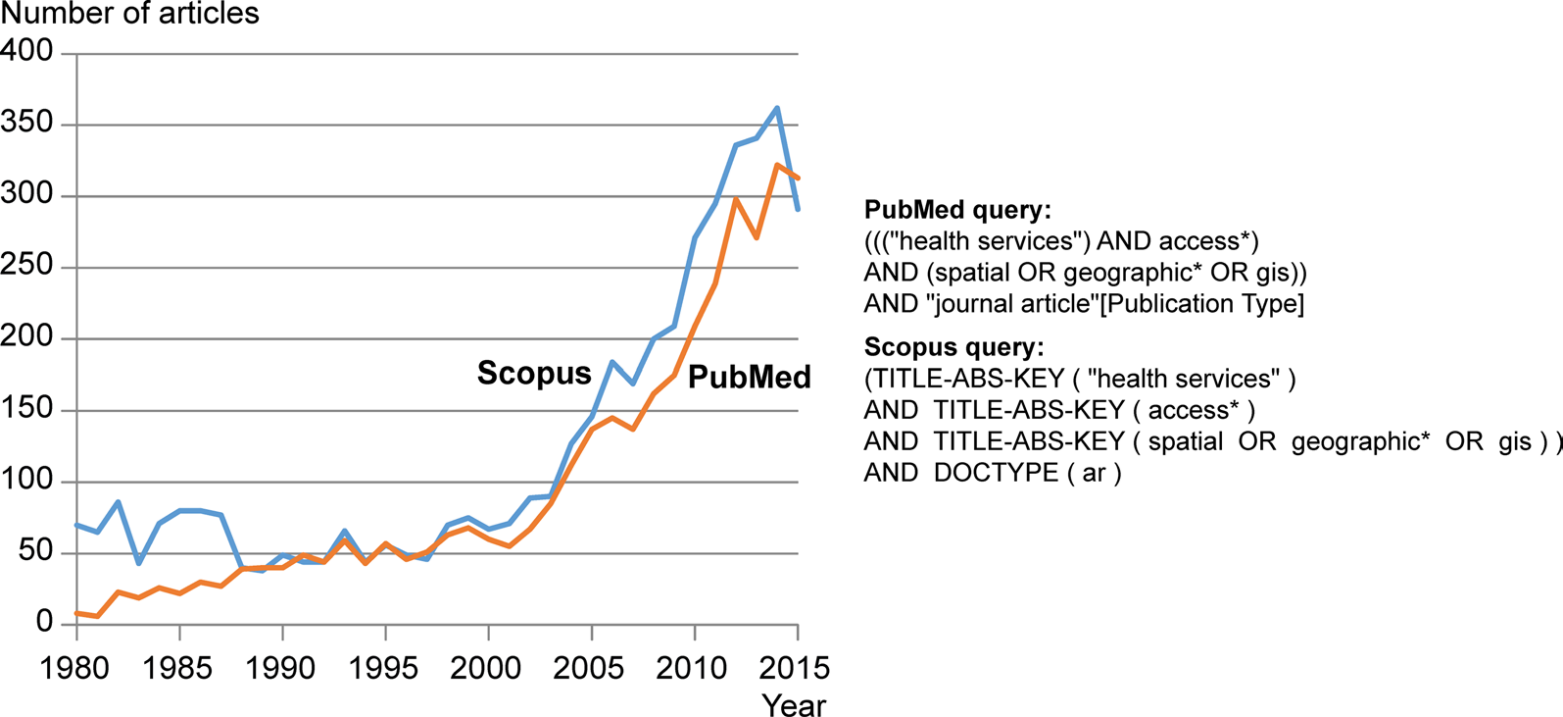
Number of journal articles published related to geographical accessibility, 1980–2015

The geographical accessibility of services (e.g. health services, food stores, etc.) is an important issue in health geography, spatial epidemiology and public health. Since the 2000s, moreover, a growing number of articles have been published on this topic (Fig. 1). In the wake of the seminal article by Penchansky and Thomas [1], it has generally been agreed that the concept of access is multidimensional and can be defined in terms of affordability, acceptability, availability and spatial accessibility. Other scholars also note that this notion can be defined according to two dimensions: potential or revealed, and spatial or aspatial [1–4]. Potential accessibility considers the probable utilization of services, given the population size and its demographics, while revealed accessibility concerns the actual use of services. Spatial access analyzes the importance of spatial separation between supply and demand as a barrier or a facilitator, and aspatial access focuses on non-geographical barriers or facilitators [2, 5]. Consequently, the notion of access to health services encompasses four major categories: revealed spatial access, revealed aspatial access, potential spatial access, and potential aspatial access [1]. This study focuses on potential spatial access, which refers to the ease with which residents of a given area can reach services and facilities [6].

The deployment of potential spatial access measures requires the specification of a set of four parameters, namely: (1) a spatial unit of reference for the population, i.e. a definition of residential areas (e.g. census tracts); (2) an aggregation method, i.e. to account for the distribution of population in the residential area; (3) a measure of accessibility; and (4) a type of distance to be used in computing the accessibility measures selected [6]. As shown in a previous study [6], the choice of these parameters is likely to generate different results, which could potentially lead to significant measurement errors. For example, for the Montreal metropolitan area, this study has shown that potential spatial access varies a great deal for 10% of census tracts, and mainly for those located in suburban areas, according to the type of distance used, and according to the aggregation method [6]. However, no study has attempted to simultaneously evaluate the impact of these various parameters in order to identify their respective importance in the evaluation of potential spatial access; this is what we now propose to do, with the help of sensitivity and uncertainty analyses.

Concretely speaking, the objective of this paper is to revisit that previous study [6] by adding three important improvements. It is first a matter of revisiting the comparison of the types of distance by including four new time-distances according to the mode of transportation used: walking, cycling, public transit and car. Indeed, since the advent of general transit feed specification (GTFS) files, more and more studies on the access to services have been based on shortest network time (by public transit) [7–13]. Other recent research, although rarer, also looks at bicycle accessibility [14]. The second improvement involves evaluating aggregation errors by including another aggregation method based on the utilization of a land use map. Thirdly, other accessibility measures that have been proposed in recent years, such as the two-step floating catchment area (2SFCA) method and its variants, have been added.

### Evaluating potential spatial access to services and facilities in residential areas: specifying four parameters

#### Spatial unit of reference and aggregation methods

Selecting the appropriate spatial unit of analysis, i.e. the operational definition for residential areas, is critical for minimizing aggregation errors [6, 15]. Aggregation error arises from the distribution of individuals around the centroid of spatial units [15]. In the urban context, as spatial units vary in size from smaller areas, such as census blocks, to larger ones, such as census tracts, the accessibility measured for smaller units is less subject to aggregation error than that measured for larger spatial units [15].

As indicated by Hewko et al. [15], the census tract is often selected for several reasons. First, detailed socioeconomic, socio-demographic, and housing data are available at the census tract level, which is not always the case at finer levels such as dissemination areas or census blocks. So, if we want to place accessibility measures in relation to socioeconomic variables, by using either classic or multilevel regressions, the census tract remains a highly relevant choice. Secondly, census tracts include on average about 5000 inhabitants and are relatively homogeneous on the socioeconomic level and from the point of view of housing. They consequently represent a division at the neighbourhood level that is widely used by urban planners and public health experts. Nonetheless, using the census tract requires that we then apply aggregation methods so as to limit errors in the measurement of potential spatial access.

**Fig. 2:**
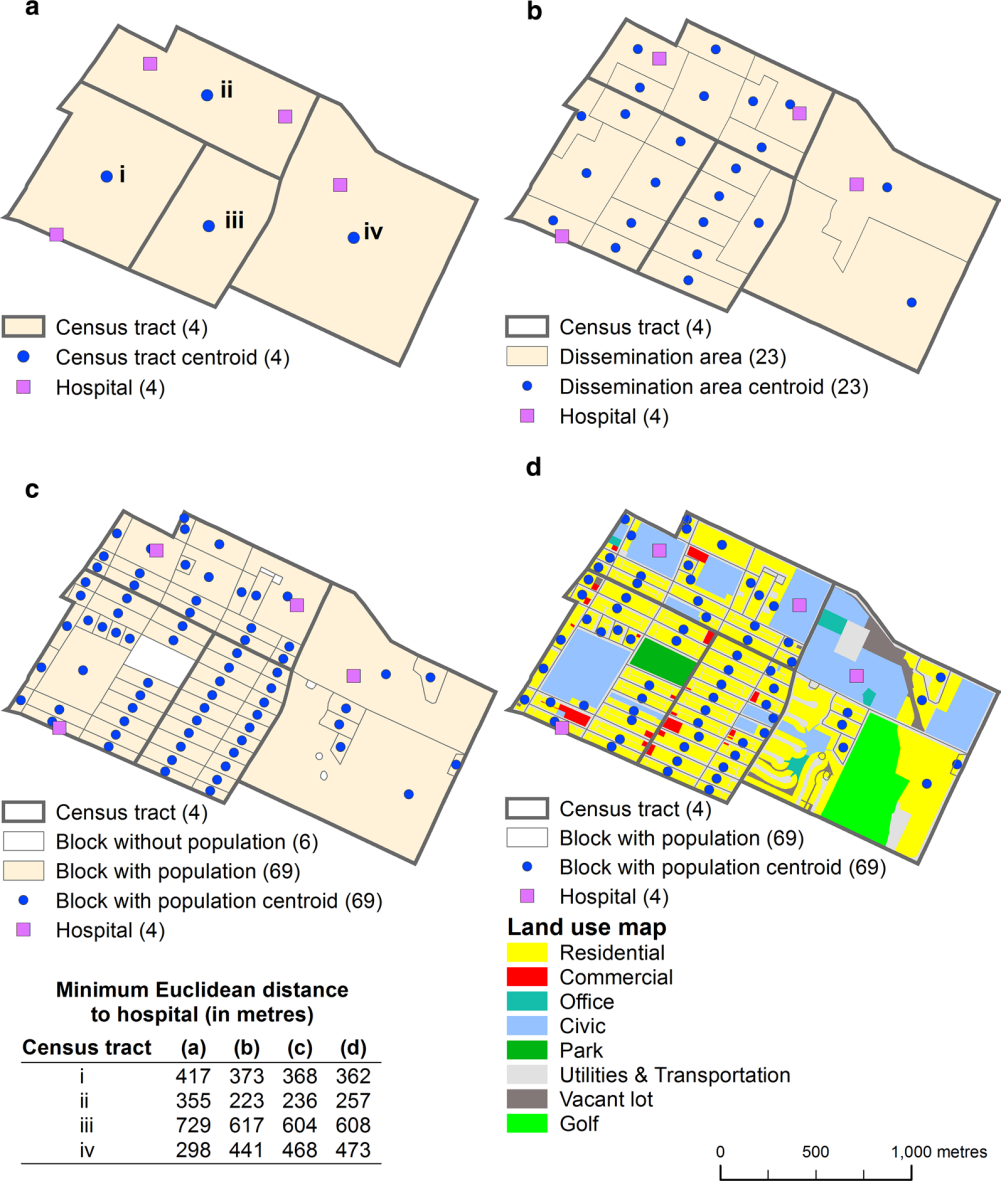
Choosing the spatial unit of reference for calculating distances and error aggregation. **a** Census tract. **b** Census tract versus dissemination area. **c** Census tract versus block. **d** Census tract versus block centroid adjusted with the land use map. *Note* *Number in parenthesis* indicates the number of spatial units

To evaluate the potential spatial access to a health service for a population living in a residential area, e.g. a census tract, several methods can be used [6, 15, 16]. The first method consists in computing the distance between the centroid of the census tract and the service (Fig. 2a). This method shows the inappropriateness of ignoring the spatial distribution of the population inside the census tract. The second method consists in calculating the population-weighted mean centre of the census tract (Eq. 1) and then evaluating the distance between this new location and the service. Toward this end, smaller spatial units entirely contained within the census tracts can be used, such as dissemination areas, census blocks, postal codes or buildings. This method accounts for the spatial distribution of the population inside the census tract in order to minimize aggregation error.

1 $$\left( {\overline{{x_{i} }} ,\overline{{y_{i} }} } \right) = \left( {\frac{{\sum\nolimits_{b \in i} {w_{b} x_{b} } }}{{\sum\nolimits_{b \in i} {w_{b} } }},\frac{{\sum\nolimits_{k \in i} {w_{b} y_{b} } }}{{\sum\nolimits_{b \in i} {w_{b} } }}} \right)$$

where *w*_*b*_ represents the total population of spatial unit *b* completely within census tract *i* (i.e. dissemination area or census block or postal code) and *x*_*b*_, *y*_*b*_ are the Cartesian coordinates of the spatial unit *b*.

The third method consists in computing the distance between the services and each centroid of spatial units completely within census tracts, and then calculating the average of these distances weighted by the total population of each unit. For example, this operation is shown based on dissemination areas and blocks contained within census tracts in Fig. 2b, c.

The latter approach enhances the preceding one. It is a matter of calculating the accessibility measures on the level of blocks contained within census tracts, and then computing the average weighted by the population. However, the centroids of the blocks are first adjusted by using dasymetric mapping methods designed to locate the areas where the population in a given spatial unit lives (e.g. census tract, dissemination area, block) [16, 17]. This approach requires the use of either satellite images [16], or land use or cover maps [17]. The basic principle involves creating a binary mask separating residential areas (1) from non-residential areas (0). For example, as illustrated in Fig. 2d, a land use map was employed to identify the residential portion of each block (the category in yellow). A comparison of Fig. 2c, d shows that the location of the block centroids is then more precise. Compared with the previous methods, this latter method is more accurate because it more exactly accounts for the distribution of the population inside the census tract.

#### Accessibility measures

Since 2000, a number of literature reviews have been published on the accessibility of health services [6, 18–22]. They show that the five most commonly used measures of the accessibility of health services are: (1) the distance to the closest service [e.g. 23–25]; (2) the number of services within *n* metres or minutes [e.g. 26, 27]; (3) the mean distance to the *n* closest services [e.g. 28]; (4) gravity models [e.g. 2, 29, 30]; and (5) two-step floating catchment area (2SFCA) methods [2, 31–34]. Table 1 synthesizes various approaches for conceptualizing and measuring different dimensions of potential spatial access [5, 28, 35].

**Table 1:** Approaches for conceptualizing and measuring the potential spatial access to services and facilities for residential areas. Adapted from [5, 28, 35]

Conceptualization	Accessibility measures
Immediate proximity or minimum travel time or distance	The distance between a location and the closest facility
Availability provided by the immediate surroundings or cumulative opportunity	The number of facilities within a given distance from a point of origin
Average cost to reach all destinations	The average distance between a location and all facilities
Average cost to reach diversity	The average distance between a location and *n* facilities
Accessibility according to proximity and availability	Gravity models, two-step floating catchment area (2SFCA) methods

The first three measures are only based on the supply of services. The most often used measure is clearly the distance to the closest health service (e.g. nearest hospital or medical clinic). It allows one to evaluate the immediate proximity to the health services. If the most accurate aggregation method detailed above is selected, i.e. an aggregation method based on the population-weighted mean of the accessibility measure for block centroids (adjusted with the land use map) within census tracts, this accessibility measure can be written as:

2 $$A_{i}^{a} = \frac{{\sum\nolimits_{b \in i} {w_{b} \left( {\hbox{min} \left| {d_{bs} } \right|} \right)} }}{{\sum\nolimits_{b \in i} {w_{b} } }}$$

where a weaker value of *A*_*i*_^*a*^ implies better accessibility, *w*_*b*_ is the total population of census block *b* completely within census tract *i* and *d*_*bs*_ is the distance between census block *b* and service *s.*

The second accessibility measure—the number of services within *n* metres or minutes—refers to the cumulative opportunity, or, in other words, to the availability provided by the immediate surroundings:

3 $$A_{i}^{b} = \frac{{\sum\nolimits_{b \in i} {w_{b} \sum\nolimits_{j \in S} {S_{j} } } }}{{\sum\nolimits_{b \in i} {w_{b} } }},$$

where a larger value of *A*_*i*_^*b*^ implies better accessibility, *w*_*b*_ is defined as previously indicated, *S* represents all services in the study area, and *S*_*j*_ is the number of services within *n* metres or minutes of census block centroid *b* (with *S*_*j*_ = 1 where *d*_*bs*_ ≤ *n* and *S*_*j*_ = 0 where *d*_*bs*_ > *n*).

To evaluate the average cost to reach diversity, the mean distance to the *n* closest services is generally used. For example, in a study on food deserts [36], the mean distance to the three closest different chain-name supermarkets is used as a proxy of variety in terms of food and prices. For this measure, a weaker value implies better accessibility:

4 $$A_{i}^{c} = \frac{{\sum\nolimits_{b \in i} {w_{b} \sum\nolimits_{s} {\frac{{d_{bs} }}{n}} } }}{{\sum\nolimits_{b \in i} {w_{b} } }},$$

where *w*_*b*_ is defined as previously indicated, *d*_*bs*_ represents the distance between spatial unit centroid *b* and service *s* (*d*_*bs*_ is sorted in ascending order), and *n* is the number of closest services to be included in the measure.

However, the three measures described above are only based on the supply of services. Now, as mentioned by several authors, as mentioned by several authors [2, 29, 30], the potential spatial access to health care depends on both the location of the supply of health services and the residential location of potential health users (demand). Two types of measures allow for take these two dimensions into account (supply and demand): i.e. gravity models [e.g. 2, 29, 30] and two-step floating catchment area (2SFCA) methods. In including an accurate aggregation method, the gravity models can be written as:

5 $$A_{i}^{d} = \frac{{\sum\nolimits_{b \in i} {\sum\nolimits_{j = 1}^{s} {\frac{{S_{j} d_{bj}^{ - \alpha } }}{{V_{j} }}} } }}{{n_{i} }}\quad {\text{with}}\quad V_{j} = \sum\limits_{b}^{m} {w_{b} } d_{bj}^{ - \alpha } ,$$

where *A*_*i*_^*d*^ is the mean value of potential gravity for census tract *i* (a larger value implies better accessibility), *n* and *s* are respectively the number of census blocks and of services in the study area, *S*_*j*_ is the weight given to service *s* such as its size (e.g. number of beds in a hospital) (“supply side”), *V*_*j*_ is the potential population (“demand side”), *α* represents the friction parameter (usually 1, 1.5 or 2), and, finally, *n*_*i*_ is the number of blocks within census tract *i.*

The 2SFCA method is a fairly recent one; it was proposed in 2003 by Luo and Wang [2, 37], based on the work of Radke and Mu [38]. As its name indicates, it includes two steps. The first step assigns an initial ratio to each health service, which takes the following form:

6 $$R_{j} = \frac{{S_{j} }}{{\sum\nolimits_{{k \in \left\{ {d_{kj} \le d_{0} } \right\}}} {P_{k} } }}$$

where *R*_*j*_ represents the supply-to-demand ratio within catchment area *d*_0_, *d*_*kj*_ is the distance between spatial unit census tract centroid *k* and health service *j*, *d*_0_ is the threshold distance or travel time (e.g. one kilometre or 30 min), *S*_*j*_ and *P*_*k*_ are respectively the supply capacity (e.g. number of medical clinics or number of beds in a hospital) at location *j* and the demand at location *k* that falls within catchment area *j*. Note that *P*_*k*_/1000 could also be used in order to obtain an initial ratio for 1000 inhabitants within the catchment area.

In the second step, for each demand location *i* (census tract centroid), we search all supply locations *j* within the threshold distance *d*_0_ from *i* and sum up the initial supply-to-demand ratios *R*_*j*_:

7 $$A_{i}^{e} = \sum\limits_{{j \in \left\{ {d_{ij} \le d_{0} } \right\}}} {R_{j} } = \sum\limits_{{j \in \left\{ {d_{ij} \le d_{0} } \right\}}} {\left( {R_{j} = \frac{{S_{j} }}{{\sum\nolimits_{{k \in \left\{ {d_{kj} \le d_{0} } \right\}}} {P_{k} } }}} \right)}$$

where a larger value of *A*_*i*_^*e*^ implies better accessibility for census tract *i*.

Many authors have suggested improvements to the 2SFCA method in order to remedy two limitations [4, 5, 8, 31, 33, 39–42]. Firstly, in its initial form, the 2SFCA method assumes that the population (*P*_*k*_) inside the catchment area (where *d*_*kj*_ < *d*_0_) has the same accessibility regardless of the distance separating this population from the health service. Secondly, beyond the threshold distance (*d*_0_), the accessibility is null. Luo and Qi [31] have thus proposed the enhanced two-step floating catchment area (E2SFCA) method, which is now widely used [42–51]. These authors then divide the catchment area into three zones: 0–10 min (*d*_1_), 10–20 min (*d*_2_), and 20–30 min (*d*_3_) (Eqs. 8, 9). For each of these three zones, it is then possible to apply a weighting (*W*_*k*_) calculated by using a Gaussian function:

8 $$R_{j} = \frac{{S_{j} }}{{\sum\nolimits_{{k \in \left\{ {d_{kj} \in D_{r} } \right\}}} {P_{k} W_{r} } }} = \frac{{S_{j} }}{{\sum\nolimits_{{k \in \left\{ {d_{kj} \in d_{1} } \right\}}} {P_{k} W_{1} } + \sum\nolimits_{{k \in \left\{ {d_{kj} \in d_{2} } \right\}}} {P_{k} W_{2} } + \sum\nolimits_{{k \in \left\{ {d_{kj} \in d_{3} } \right\}}} {P_{k} W_{3} } }}$$

9 $$A_{i}^{e} = \sum\limits_{{j \in \left\{ {d_{ij} \le d_{r} } \right\}}} {R_{j} } = \sum\limits_{{j \in \left\{ {d_{ij} \in d_{1} } \right\}}} {R_{j} W_{1} } + \sum\limits_{{j \in \left\{ {d_{ij} \in d_{2} } \right\}}} {R_{j} W_{2} } + \sum\limits_{{j \in \left\{ {d_{ij} \in d_{3} } \right\}}} {R_{j} W_{3} }$$

where *W*_1_, *W*_2_, *W*_3_ = 1.00, 0.68 and 0.22 with a slow step-decay function or 1.00, 0.42 and 0.09 with a fast step-decay function. Also, some authors add a fourth zone of 30–60 min, especially when the study area includes rural areas [39, 52]. The weightings are then: *W*_1_, *W*_2_, *W*_3_, *W*_4_ = 1.00, 0.80, 0.55 and 0.15 with a slow step-decay function or 1.00, 0.60, 0.25 and 0.05 with a fast step-decay function. Note that the values of the radii can be modified according to the geographical context. For example, Dewulf et al. [23]—who analyze the accessibility of primary health care not in an urban context but on the scale of an entire country (Belgium)—use radii of 1, 2, 5 and 10 km.

As mentioned by McGrail [32], some scholars criticize the fact that the weightings are constant within each radius and advocate using a continuous weighting function: W = 1 for the first radius (0–10 min, for example); W = 0 when the distance is >60 min; and *W* = ((60 − *d*)/(60 − 10))^1.5^ for the radius of 10–60 min. Finally, to reduce aggregation errors, Bell et al. [33] recommend a 3SFCA: it is a matter of calculating the 2SFCA or the E2SFCA at a fine scale (e.g. dissemination areas and blocks within census tracts), and then calculating the mean per census tract. Consequently, by applying the most accurate aggregation method, the E2SFCA can thus be formulated with four radii or with a continuous weighting function:

10 $$\begin{aligned} R_{j} & = \frac{{S_{j} }}{{\sum\nolimits_{{b \in \left\{ {d_{bj} \in d_{1} } \right\}}} {P_{k} W_{1} } + \sum\nolimits_{{b \in \left\{ {d_{bj} \in d_{2} } \right\}}} {P_{k} W_{2} } + \sum\nolimits_{{b \in \left\{ {d_{bj} \in d_{3} } \right\}}} {P_{k} W_{3} + \sum\nolimits_{{b \in \left\{ {d_{bj} \in d_{4} } \right\}}} {P_{k} W_{4} } } }}\,\,{\text{or}} \\ R_{j} & = \frac{{S_{j} }}{{\sum\nolimits_{{b \in \left\{ {d_{bj} \le d_{0} } \right\}}} {P_{k} W_{bj} } }} \\ \end{aligned}$$

11 $$\begin{aligned} A_{i}^{e} & = \frac{{\sum\nolimits_{b \in i} {\sum\nolimits_{{j \in \left\{ {d_{bj} \in d_{1} } \right\}}} {R_{j} W_{1} } } }}{{n_{i} }} + \frac{{\sum\nolimits_{b \in i} {\sum\nolimits_{{j \in \left\{ {d_{bj} \in d_{2} } \right\}}} {R_{j} W_{2} } } }}{{n_{i} }} + \frac{{\sum\nolimits_{b \in i} {\sum\nolimits_{{j \in \left\{ {d_{bj} \in d_{3} } \right\}}} {R_{j} W_{3} } } }}{{n_{i} }} \\ & \quad + \frac{{\sum\nolimits_{b \in i} {\sum\nolimits_{{j \in \left\{ {d_{bj} \in d_{4} } \right\}}} {R_{j} W_{4} } } }}{{n_{i} }}{\mkern 1mu} {\mkern 1mu} \quad {\text{or}}\quad {\mkern 1mu} {\mkern 1mu} \frac{{\sum\nolimits_{b \in i} {\sum\nolimits_{{j \in \left\{ {d_{bj} \le d_{0} } \right\}}} {R_{j} } } }}{{n_{i} }} \\ \end{aligned}$$

where *n*_*i*_ is the number of blocks within the census tract; *W*_1_, *W*_2_, *W*_3_, *W*_4_ = 1.00, 0.80, 0.55 and 0.15 with a slow step-decay function or 1.00, 0.60, 0.25 and 0.05 with a fast step-decay function; and *W*_*bj*_ is the weight for block *j* with a continuous weighting function. This last parameter can be calculated as follows:

- if *d*_*ij*_ < 10 then *W*_*bj*_ = 1; if *d*_*ij*_ > 10 and *d*_*ij*_ ≤ 60 then *W*_*bj*_ = ((60 − *d*)/(60 − 10))^1.5^; if *d*_*ij*_ > 60 then *W*_*bj*_ = 0.

#### Types of distance

**Fig. 3:**
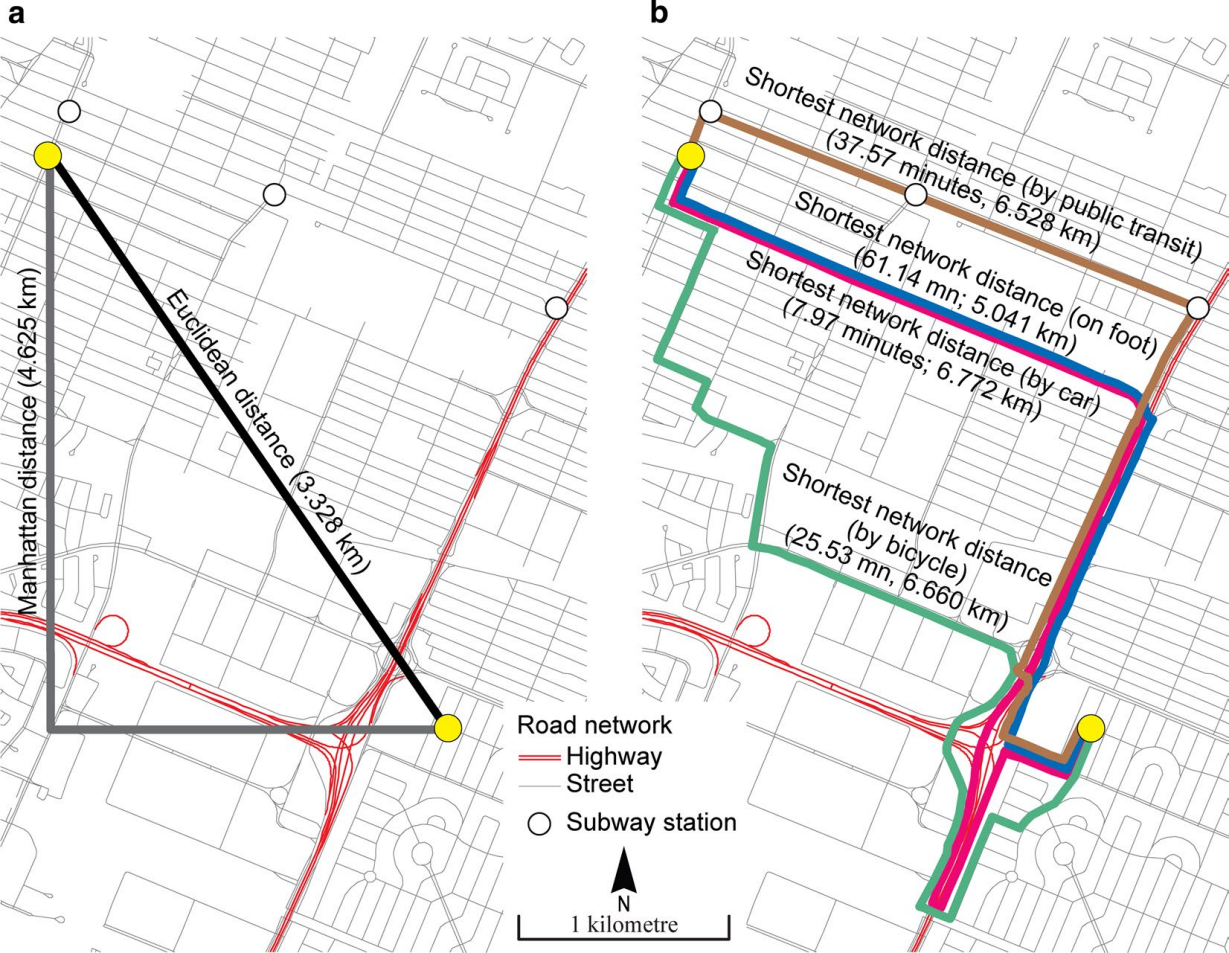
Types of distance. **a** Cartesian distances. **b** Network distances

Six types of distance can be used to calculate accessibility measures: Euclidean distance (straight-line), Manhattan distance (distance along two sides of a right-angled triangle opposed to the hypotenuse), and shortest network time distances according to the mode of transportation used (on foot, by car, by bicycle, or by public transit) (Fig. 3) [28, 31].

### Study objectives

In this paper, we investigate differences in results when the geographical accessibility of selected health care services for residential areas (census tracts) is computed by using three parameters: (1) six types of distance, (2) four aggregation methods, and (3) fourteen accessibility measures. The specific objectives are to: (1) Compare the types of distance; (2) Estimate aggregation errors for several accessibility measures; and (3) Measure how the assessment of potential spatial access varies according to these three parameters.

## Data and methods

### Study area and health services

**Fig. 4:**
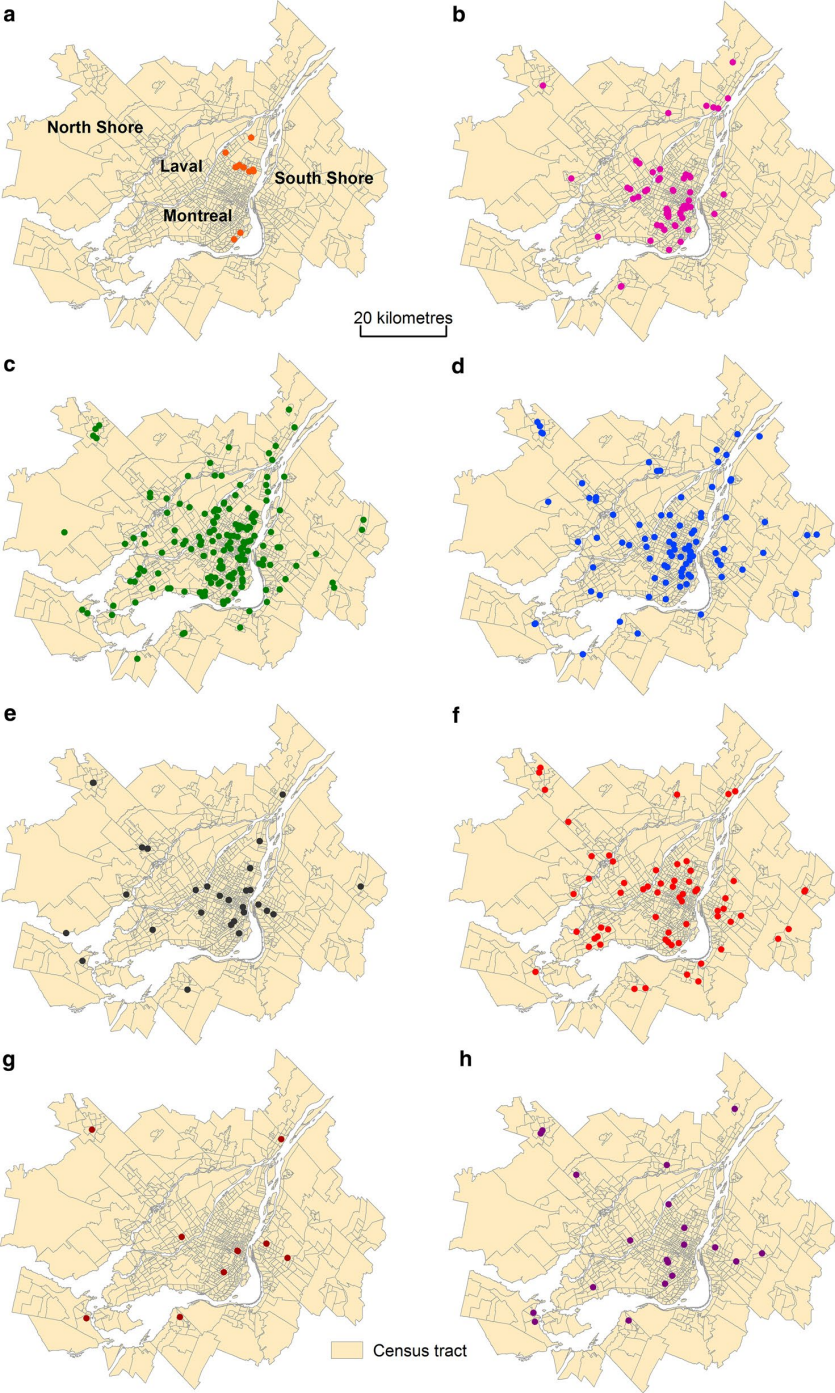
Categories of health services for the Montreal CMA, 2015. **a** Psychiatric care hospitals (N = 14). **b** General and specialized care hospitals (N = 62). **c** Long-term care facility (N = 176). **d** Local community service centre (N = 90). **e** Child and youth protection centre (N = 29). **f** Rehabilitation center for persons with intellectual disabilities (N = 67). **g** Rehabilitation center for hearing impaired persons (N = 10). **h** Rehabilitation center for physically impaired persons (N = 20) *Source* Quebec Ministry of Health and Social Services

**Fig. 5:**
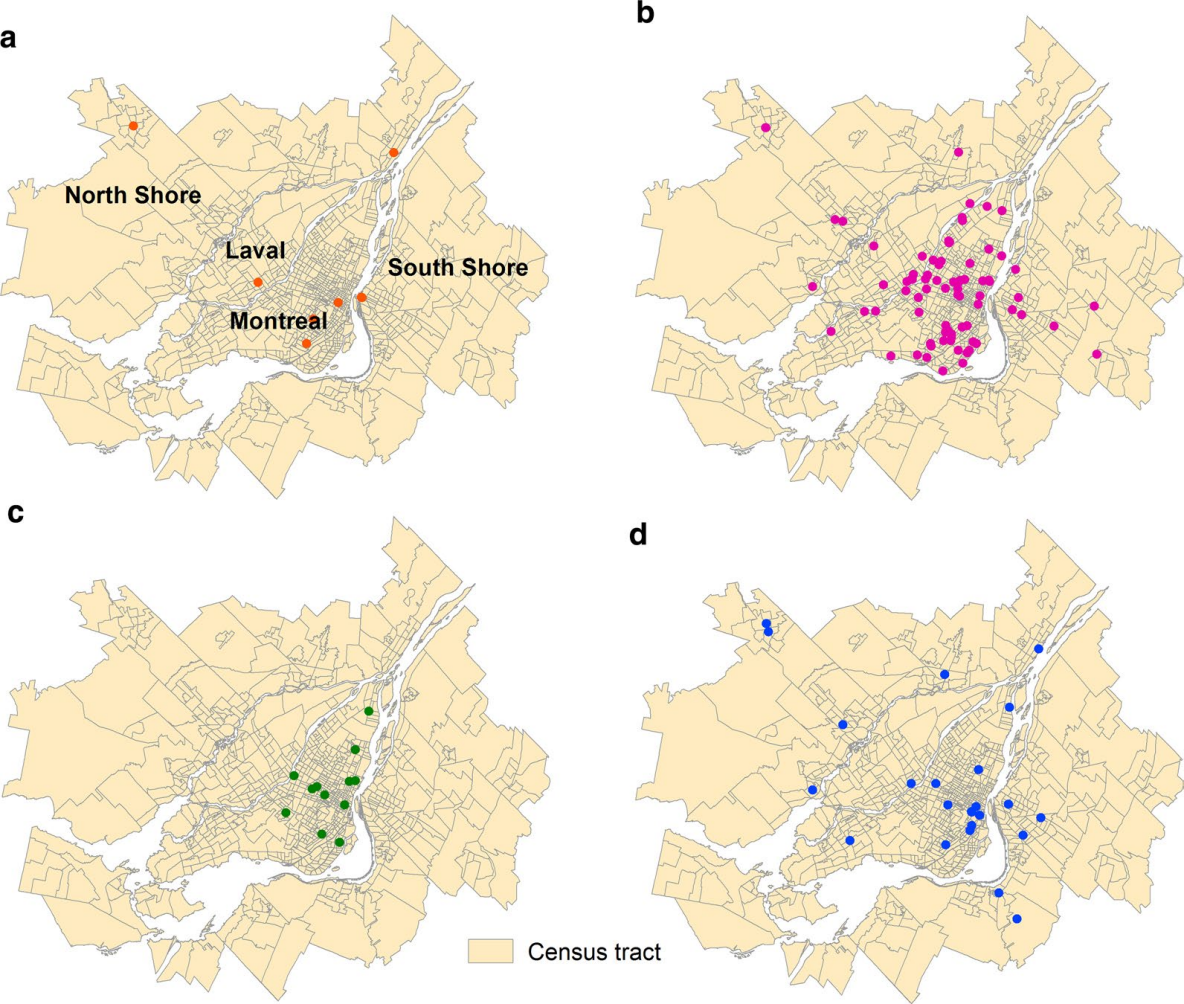
Categories of health services for the Montreal CMA, 2015. **a** Rehabilitation center for visually impaired persons (N = 8). **b** Rehabilitation center for young people with adaptation difficulties (N = 81). **c** Rehabilitation center for young mothers with adaptation difficulties (N = 12). **d** Rehabilitation center for alcohol, drug and other dependencies (N = 25). *Source* Quebec Ministry of Health and Social Services

This study focuses on the territory served by the regional transit authority for the Montreal area, which had a population of about 3.8 million in 2011. The extent of this territory is very similar to that of the Montreal census metropolitan area (CMA). The study area is divided into 904 census tracts, 6167 dissemination areas and 27,126 blocks with respective average population sizes of 4170, 611 and 139 inhabitants, as defined by Statistics Canada. A total of 594 health services grouped into twelve categories were integrated into geographic information systems (ArcGis) (Figs. 4, 5). Note that a street address can include several categories of health services. In the end, this ultimately produces 535 geographical locations for these health services; they have all been precisely geocoded from the centroid of the building. This spatial dataset was provided by the Quebec Ministry of Health and Social Services.

### Computing the six types of distance

#### Cartesian distances

Euclidean and Manhattan distances can easily be computed by using geographic coordinates:

12 $$d_{ij} = \sqrt {(x_{i} - x_{j} )^{2} + (y_{i} - y_{j} )^{2} } ,$$

13 $$d_{ij} = \left| {x_{i} - x_{j} } \right| + \left| {y_{i} - y_{j} } \right|,$$

where *x*_*i*_, *y*_*i*_, *x*_*j*_, *y*_*j*_ are the Cartesian coordinates of points *i* and *j* with a plane projection.

#### Shortest network distance (by car)

To calculate trips as though made by car, we used the Adresses Québec (AQ Directions) [53] road network—which includes the speed limits and directions of traffic for all road and street segments in the province of Quebec. Based on the length of the road or street segment and the speed limit on that road or street, the cost in minutes to travel over each segment of the road network can then be calculated [54]:

14 $$T_{mn} = \frac{{L_{ft} *60}}{{S_{mph} *5280}}\,\,\,\,{\text{or}}\,\,\,\,T_{mn} = \frac{{L_{m} *60}}{{S_{kmh} *1000}},$$

where *T*_*mn*_ is the cost in minutes to travel over the road or street segment, *L*_*ft*_ and *L*_*m*_ are the length of the segment in feet and metres respectively, and *S*_*mph*_ and *S*_*kmh*_ are the speed limits in miles/h and km/h.

#### Shortest network distance (on foot)

**Fig. 6:**
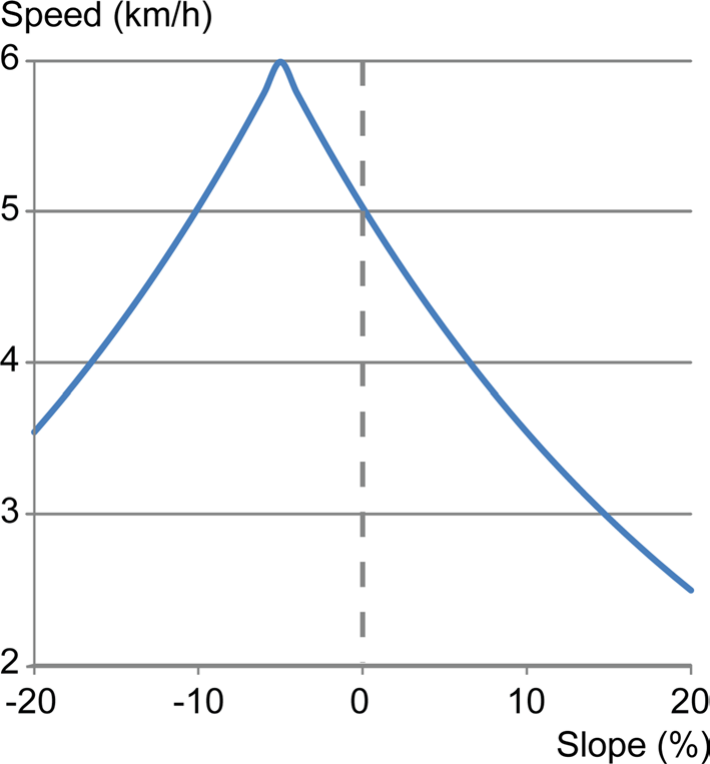
Walking speed according to Tobler’s hiking function

The modeling of the network for travel on foot is also based on the Adresses Québec road network. Compared with the modeling of the network by car, a restriction was added on segments of highway where pedestrians are not allowed, whereas the direction of traffic was not used as a restriction. Moreover, the elevation of each junction of the network was extracted from a digital elevation model at a resolution of 3 m. Based on these elevation data for the junctions, it is then possible to calculate the walking speed over the road or street segment (*W*_*kmh*_) by using the classic Tobler’s hiking function [55]:

15 $$W_{kmh} = 6e^{{ - 3.5\left| {\frac{dh}{dx} + 0.05} \right|}}$$

where *dh* is the difference in elevation between the start and end nodes of the road or street segment, and *dx* is the segment’s length. When the slope is equal to 0 (flat terrain), the walking speed is equal to 5 km/h (Fig. 6). By applying Eqs. (14) and (15) as described above, it is then possible to estimate the cost in minutes of foot travel for each segment from the start node to the end node, and vice versa.

#### Shortest network distance (by bicycle)

The cycling network was modeled by combining several sources of data on bicycle paths obtained from the municipalities of Montreal, Longueuil and Laval and OpenCycleMap. This network was then merged with the Adresses Québec road network. Elevation data were again used to calculate the slope for each segment of the network. There is currently no consensus on cyclists’ average travel speed in urban areas. For example, Jensen et al. [56] found an average speed of 14.5 km/h for cyclists in Lyon (France), whereas Parkin and Rotheram [57] in Leeds (UK) obtained an average speed of 21.6 km/h. However, other studies regularly suggest values of about 16 km/h [58–61], the average value used by Google Maps,^1^ which was also selected for this study. Other authors have shown that speed varies according to the slope, length of segment, type of cycling infrastructure and type of bicycle. With the help of a regression model, Parkin and Rotheram [57] have thus estimated the impact of slope on travel speeds: that is, 0.86 km/h for each percentage of downhill gradient and −1.44 km/h for each percentage of uphill gradient. El-Geneidy et al. [58] looked at the impact of infrastructures on cyclists’ travel speeds in Minneapolis (USA). They concluded that, all other things being equal, only off-street bicycle paths have a significant and positive impact on speed (1.14 km/h) and that each kilometre of the segment length is associated with a 0.32 km/h increase in speed. Consequently, we modeled cyclists’ travel speeds (*C*_*kmh*_) on each segment as follows:

16 $$\begin{aligned} C_{kmh} & = 16 + \left\{ {\begin{array}{*{20}l} {\left( {\left| {s_{i} } \right|* - 1.44} \right)} \hfill & {if\,\, s_{i} > 0} \hfill \\ {\left( {\left| {s_{i} } \right|*0.86} \right)} \hfill & {if\,\, s_{i} < 0} \hfill \\ \end{array} } \right. + l_{i} *0.32 \\ & \quad + \,\left\{ {\begin{array}{*{20}l} {1.14} \hfill & {if\,\,t_{i} = off\,\,street} \hfill \\ 0 \hfill & {otherwise} \hfill \\ \end{array} } \right. \\ \end{aligned}$$

where *s*_*i*_ is the percentage of slope on the segment from the start to the end node or vice versa, *l*_*i*_ is the segment length in kilometres, and *t*_*i*_ is the type of cycling infrastructure.

#### Shortest network distance (by public transportation)

As done by Faber et al. [62] and Hadas [63], general transit feed specification (GTFS) files are used to calculate travel times with public transit. GTFS data covering all of our study area were obtained from the Agence Métropolitaine de Transport (AMT) (regional transit authority for the bus, metro and commuter train network). These data were integrated into ArcGIS and combined with the pedestrian network by using the Add GTFS to a Network Dataset^2^ tool. Since travel times can vary according to the time of departure, especially in outlying municipalities where commuter trains and buses run far less frequently than in central neighbourhoods, we calculated 13 distance matrices: that is, for Monday departures every 10 min from 7:00 a.m. to 9:00 a.m. We then selected the minimum travel time for each of the 13 trips between census spatial units (census tracts, dissemination areas and blocks) and health services. This ensured that the travel times would not be overestimated, especially for trips to or from the suburbs.

### Comparing distance types

To explore variations in results according to distance type (Objective 1), we calculate the six distance types—Euclidean, Manhattan, and shortest network time distances (on foot, by car, by bicycle, or by public transit)—between the 535 health services and the centroids of census tracts (n = 904), dissemination areas (n = 6167) and blocks (n = 27,126) and block centroids adjusted with a land use map. In total, close to 197 million distances are computed (Table 2), with a Python code for Euclidean and Manhattan distances, and with the Network Analyst Extension of ArcGIS (version 10.3) for the four shortest network time distances.

**Table 2:** Distances calculated between health services and spatial units

Spatial units (origins)		Health services (destinations)	Types of distance^a^	Distances calculated
Type	N			
Census tract centroids	904	535	6	2,901,840
Dissemination area centroids	6167	535	6	19,796,070
Block centroids	27,126	535	6	87,074,460
Block centroids adjusted with the land use map	27,126	535	6	87,074,460
Total	61,323	535	6	196,846,830

^a^Euclidean, Manhattan, shortest network time (on foot), shortest network time (by car), shortest network time (by bicycle), and shortest network time (by public transit)

Once these distance types are computed, correlation analyses are performed globally and locally across all the census tracts, dissemination areas and blocks matrices. First, the global analysis, which yields one value for the study area as a whole, allows us to assess the degree of correlation between the four distance types. Then, we examine correlations between the four distances for each spatial unit centroid and the 535 health service locations. This local analysis stage yields one mappable value for each census tract, dissemination area and block and allows us to identify spatial variation in the degree of correlation between the six distance types.

### Evaluating aggregation errors when measuring potential geographic access

The same approach, i.e. global and local analyses, was used to evaluate aggregation errors for several accessibility measures at the census tract level (Objective 2). To do this, we calculated 14 accessibility measures (Table 3), using six types of distance and four aggregation methods, for a total of 336 measures. Although accessibility was computed for each of the twelve categories of health services, for purposes of conciseness, results are reported only for the accessibility of general and specialized care (i.e. hospitals; n = 62) for census tracts. It is worth noting that similar patterns of correlation were observed for the other health services.

**Table 3:** List of measures of accessibility computed

	Distance type
1.	Minimum distance
2.	Average distance to all hospitals
3.	Average distance to three closest hospitals
4.	Average distance to five closest hospitals
5.	Number of hospitals within 500 m or 10 min
6.	Number of hospitals within 1000 m or 20 min
7.	Number of hospitals within 2000 m or 30 min
8.	Potential gravity model (friction parameter = 1)
9.	Potential gravity model (friction parameter = 1.5)
10.	Potential gravity model (friction parameter = 2)
11.	Two-step floating catchment area (2000 m or 30 min)
12.	Enhanced two-step floating catchment area with a slow step-decay function^a^
13.	Enhanced two-step floating catchment area with a fast step-decay function^a^
14.	Enhanced two-step floating catchment area with a gradient function

^a^The catchment area is divided into three zones: 0–500 m or 0–10 min, 500–1000 m or 10–20 min, and 1000–2000 m or 20–30 min

The global analysis involves calculating correlations between four aggregation methods: (1) the census tract centroid (CTC); (2) the population-weighted mean of the accessibility measure for dissemination areas within census tracts (WDAC); (3) the population-weighted mean of the accessibility measure for blocks within census tracts (WBL1); and (4) the population-weighted mean of the accessibility measure for blocks (adjusted with the land use map) within census tracts (WBL2).

The local analysis consists in simply calculating the absolute differences for each of the 14 accessibility measures obtained with the least accurate aggregation method (CTC) and the most accurate method (WBL2). It is then possible to calculate the univariate statistics and to map these differences.

### Sensitivity analysis (SA) and uncertainty analysis (UA)

Sensitivity and uncertainty analyses are mainly used to test the robustness of composite indicators [64, 65].

During these analyses, a number of methodological choices in fact intervene and modify the final index indicator values. So it is important to determine how sensitive the indicator is to these choices. A very unstable (i.e. highly uncertain) indicator is problematic, as it can be strongly influenced by specific methodological choices (a particular weighting, for example). Conversely, a very rigid indicator is not necessarily desirable either, because methodological choices are supposed to help to construct the index indicator, to give it meaning.

This type of analysis applies when one has a final score, obtained with the help of a model, which itself depends on several parameters. These parameters are called uncertainty factors because they can take on several different values that will alter the final score. This description makes clear the parallel with our study. Indeed, our final score is an indicator of potential spatial access, obtained by using a model that includes three uncertainty factors: the type of distance (6 choices), the aggregation method (4 choices), and the accessibility measure (14 choices). This model can then take 336 different forms; in other words, in the context of this study, there are 336 different ways of calculating a potential spatial access score for each census tract. To our knowledge, this method has never been used in this context, so that this is an original application. Indeed, by using a sensitivity analysis (SA), we can explain how each of the three parameters leads to variation in the levels of accessibility for the entire study area (Objective 3). Also, the use of an uncertainty analysis (UA) allows us to identify and map census tracts for which the 336 accessibility indicators vary the most according to the three parameters.

Before performing these two analyses, the 336 indicators need to be transformed so that they are expressed in the same units. The most common transformations are normalization on a scale of 0–1 (Eq. 17), the z-score standardization (Eq. 18), or the use of ranks (Eq. 19) [66].

17 $$I_{q,c} = \frac{{x_{q,c} - min\left( {x_{q} } \right)}}{{range\left( {x_{q} } \right)}}$$

18 $$I_{q,c} = \frac{{x_{q,c} - mean\left( {x_{q} } \right)}}{{std\left( {x_{q} } \right)}}$$

19 $$I_{q,c} = rank\left( {x_{q,c} } \right)$$

To measure the uncertainty for each census tract, we simply calculate the coefficient of variation (CV = STD/Mean) of the 336 values of the previously transformed accessibility indicators. So, for a census tract, the higher the value of the CV is, the greater the uncertainty is, or, in other words, the more the methodological choices locally impact the assessment of potential geographic access.

For the sensitivity analysis, a reference indicator must first be chosen from among the 336 indicators. We chose the least complex indicator, calculated with the following parameters: Euclidean distance, the CTC aggregation method and the closest hospital as the accessibility measure. For each of the 336 indicators, it is then possible to calculate the average of the absolute differences with respect to the reference indicator:

20 $$\overline{R}_{s} = \frac{{\mathop \sum \nolimits_{c = 1}^{n} \left| {I_{reference,c} - I_{q,c} } \right|}}{n}$$

where *n* is the number of census tracts.

One can then calculate the first order sensitivity indices (*S*_*i*_), proposed by Sobol [64, 67], i.e. the proportion of the total variance attributable to each factor (distance types, aggregation methods and accessibility measures). This method is based on Sobol’s equation of variation decomposition and is particularly suited for non-linear models. In our case, the uncertainty factors do not have a non-linear, which justifies the use of this method [68]:

21 $$S_{i} = \frac{{V_{i} }}{V\left( Y \right)} = \frac{{V_{{x_{i} }} (E_{{x_{ - i} }} (Y|X_{i} ))}}{V\left( Y \right)}$$

where *V*_*i*_ = variance explained by the uncertainty factor *X*_*i*_; *V*(*Y*) = total variance; *X* = uncertainty factors; *Y* = the overall mean shift with the reference; ${\text{E}}_{{{\text{x}}_{ - i} }} ({\text{Y}}|{\text{X}}_{\text{i}} )$ = the expected value (mean) of *Y* for all combinations of the indicator with factor *X*_*i*_ fixed to a particular modality; $V_{{x_{i} }} (E_{{x_{ - i} }} (Y|X_{i} ))$ = the variance of these means for all possible modalities of *X*_*i*_.

One can then further decompose the variance by adding second order indices that measure the proportion of the variance attributable to interactions between two parameters:

22 $$S_{ij} = \frac{{V_{ij} }}{V\left( Y \right)} = \frac{{V_{{x_{i} x_{j} }} \left( {E_{{x_{ - ij} }} \left( {Y |X_{i} ,X_{j} } \right)} \right) - V_{i} - V_{j} }}{V\left( Y \right)}$$

For example, one could evaluate the proportion of the variance that is explained by the interaction of the type of distance factor with the factor of the accessibility measure. Finally, the total effect sensitivity index for a factor is the sum of the first order and second order indices:

23 $$S_{T1} = S_{1} + S_{12} + S_{13}$$

24 $$S_{T2} = S_{2} + S_{12} + S_{23}$$

25 $$S_{T3} = S_{3} + S_{13} + S_{23}$$

where *S*_*T*1_, *S*_*T*2_, *S*_*T*3_ are the total effects for the type of distance, the aggregation method and the accessibility measure respectively. More detailed information on sensitivity and uncertainty analyses can be found especially in the work of Sobol [67], Nardo et al. [66] and Saisana et al. [64].

## Results

### Correlations between the six types of distance

**Fig. 7:**
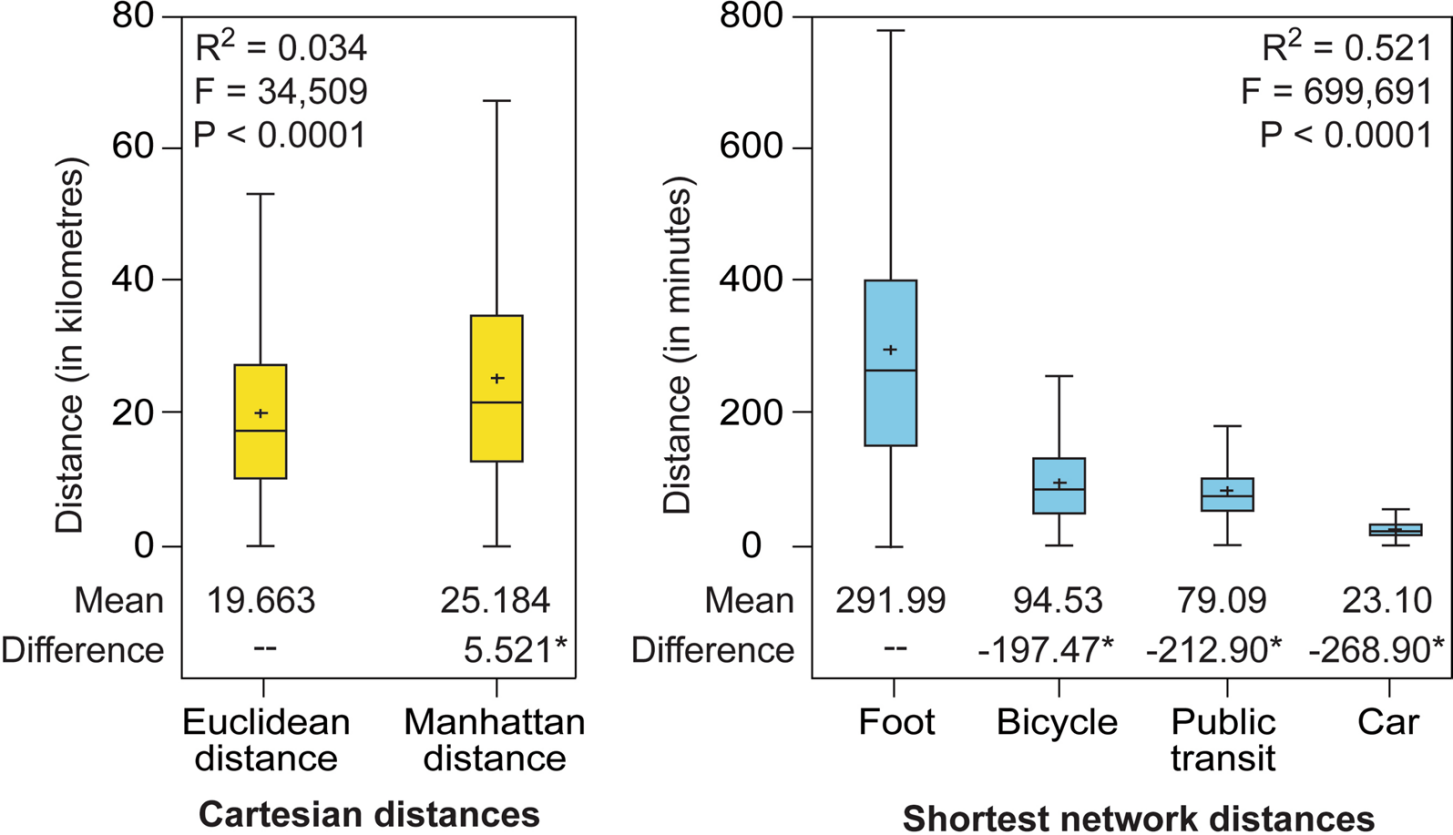
Boxplot of Cartesian and network distances between health services and census tracts. *Note* Tukey test for comparison of the mean values. *Significant difference at *P* = 0.01

Before exploring the correlations, it is relevant to analyze a few statistics for the different types of distance calculated between the 535 destinations and the 904 census tracts (n = 483,640) (Table 4). For Cartesian distances, the mean values are 19.7 km for Euclidean distance compared with 25.2 km for Manhattan distance, i.e. a significant difference of 5.5 km (*P* = 0.01) (Fig. 7). Since Manhattan distance is the length of the two sides of a right-angled triangle opposed to the hypotenuse—with the latter representing Euclidean distance—(Fig. 3a), it is therefore evident that all the univariate statistics are higher for Manhattan distance.

**Table 4:** Univariate statistics for the distances calculated between health services and census tracts

Distance type	Mean	P10	Q1	Q2	Q3	P90
Euclidean distance (km)	19,663	5565	9922	17,304	27,023	37,480
Manhattan distance (km)	25,184	7004	12,517	21,756	34,369	48,587
Shortest network time (on foot) (min)	291.99	83.80	149.67	262.20	400.96	550.37
Shortest network time (by bicycle) (min)	94.53	27.20	48.22	84.33	130.65	177.92
Shortest network time (by public transit) (min)	79.09	32.57	48.92	72.30	101.16	131.89
Shortest network time (by car) (min)	23.10	8.83	14.40	21.84	30.54	38.89

*P10* 10th percentile, *Q1* lower quartile, *Q2* median, *Q3* upper quartile, *P90* 90th percentile

Regarding the shortest network times, it is no surprise that the statistics show that the means of the trips are greater on foot (mean = 292 min), followed by trips by bicycle (mean = 95 min), public transport (mean = 79 min) and car (mean = 23 min) (Table 4; Fig. 7). In other words, compared with trips by car, the trips are, on average, 12.6 times longer on foot, 4.1 times longer by bicycle and 3.4 times longer by public transit. Another interesting result is that the value of the 10th percentile for bicycle travel times is lower than the value for public transit (27.20 vs. 32.57 min). This means that, for 10% of the fastest trips, the bicycle is 5 min faster than public transport. To put it another way, the bicycle is a very good alternative to public transit for short trips.

#### Global correlations

Table 5 presents results for global correlation coefficients between the six types of distance computed for all health service locations (n = 535). From the correlation matrices, three main results can be highlighted. First, at the metropolitan scale, independently of the type of distance used except for shortest network time by public transit, the results are globally similar as indicated by high correlation coefficient values (>0.90). Second, in comparison with Manhattan distance, Euclidean distance is most strongly correlated with all the shortest network time distances. This means that if it is impossible to compute network distances in a study focusing on geographical accessibility in the Montreal CMA, Euclidean distance seems preferable to Manhattan distance. Third, the correlations between the three shortest network times—on foot, by car, by bicycle—are very high (>0.95), but the correlations of the shortest network time (by public transit) with all other types of distance are much weaker (between 0.76 and 0.82).

**Table 5:** Global Pearson correlations between alternative types of distance

Distance	Cartesian system		Shortest network time distances			
	Euclidean	Manhattan	On foot	By car	By bicycle	By public transit
Distances between census tracts and health services (N)	483,640	483,640	483,640	483,640	483,640	483,640
Euclidean distance	1.0000					
Manhattan distance	0.9851	1.0000				
Shortest network time (on foot)	0.9719	0.9497	1.0000			
Shortest network time (by car)	0.9416	0.9148	0.9626	1.0000		
Shortest network time (by bicycle)	0.9457	0.9257	0.9716	0.9517	1.0000	
Shortest network time (by public transit)	0.7711	0.7512	0.8215	0.7978	0.8029	1.0000
Distances between dissemination areas and health services (N)	3,299,345	3,299,345	3,299,345	3,299,345	3,299,345	3,299,345
Euclidean distance	1.0000					
Manhattan distance	0.9842	1.0000				
Shortest network time (on foot)	0.9696	0.9462	1.0000			
Shortest network time (by car)	0.9386	0.9101	0.9612	1.0000		
Shortest network time (by bicycle)	0.9407	0.9194	0.9691	0.9493	1.0000	
Shortest network time (by public transit)	0.7610	0.7411	0.8115	0.7873	0.7893	1.0000
Distances between blocks and health services (N)	14,512,410	14,512,410	14,512,410	14,512,410	14,512,410	14,512,410
Euclidean distance	1.0000					
Manhattan distance	0.9826	1.0000				
Shortest network time (on foot)	0.9627	0.9373	1.0000			
Shortest network time (by car)	0.9329	0.9034	0.9594	1.0000		
Shortest network time (by bicycle)	0.9333	0.9106	0.9641	0.9459	1.0000	
Shortest network time (by public transit)	0.7377	0.7210	0.7948	0.7635	0.7670	1.0000
Distances between block centroids adjusted with the land use map and health services (N)	14,512,410	14,512,410	14,512,410	14,512,410	14,512,410	14,512,410
Euclidean distance	1.0000					
Manhattan distance	0.9826	1.0000				
Shortest network time (on foot)	0.9627	0.9374	1.0000			
Shortest network time (by car)	0.9330	0.9036	0.9594	1.0000		
Shortest network time (by bicycle)	0.9334	0.9106	0.9641	0.9460	1.0000	
Shortest network time (by public transit)	0.7390	0.7227	0.7958	0.7634	0.7676	1.0000

All coefficient values are significant at the *p* < 0.0001 level

#### Local correlations

Although the global correlations are high, they are not perfect (the values differ from one). For this reason, local variations at the intra-metropolitan scale must exist and should be examined in detail. Local Pearson correlations have been calculated from the centroids of census tracts, dissemination areas, and blocks. For purposes of simplification, we are only presenting the results for census tracts. Note that the results show similar spatial patterns for the three spatial scales.

**Fig. 8:**
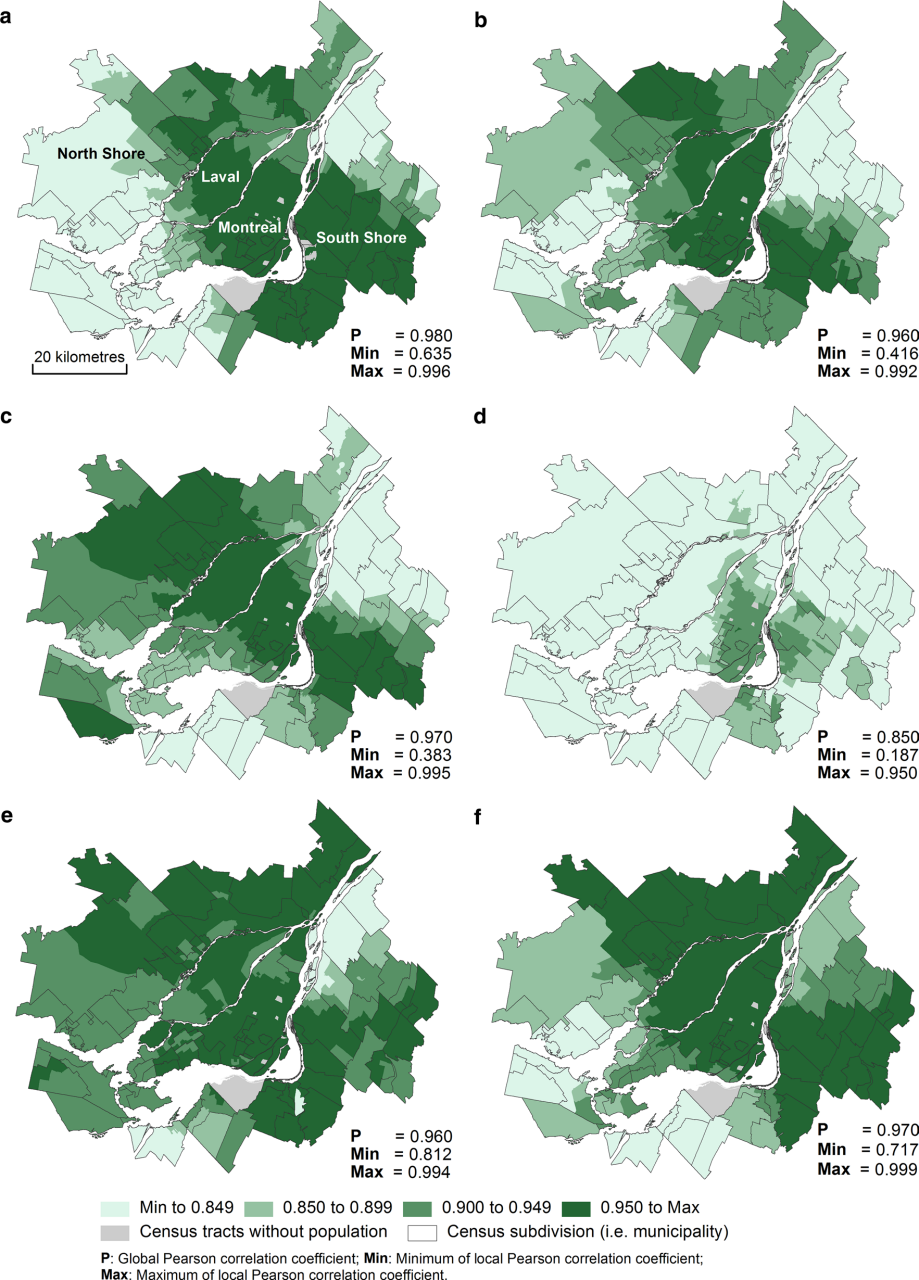
Comparing alternative types of distance between spatial units and health services using local Pearson correlations. **a** Euclidean distance versus shortest network time (on foot). **b** Euclidean distance versus shortest network time (by car). **c** Euclidean distance versus shortest network time (by bicycle). **d** Euclidean distance versus shortest network time (by public transport). **e** Shortest network time (on foot) versus shortest network time (by car). **f** Shortest network time (on foot) versus shortest network time (by bicycle)

Firstly, Fig. 8a–d presents local Pearson coefficients between Euclidean distance and the four shortest network time distances (on foot, by car, by bicycle and by public transit). The maps show that with increasing distance from the central business district, local correlations are reduced between Euclidean distance and the four shortest network time distances. For all spatial units in the centre of the Island of Montreal, the correlations are higher. For spatial units located on the periphery of the CMA, notably on the North and South shores, which are characterized by suburban areas, the correlations are weaker. It is not surprising that these results are in line with those of the previous study [6]. They also show that the local correlations between Euclidean distance and the shortest network time distance by public transit are much lower. Indeed, the strongest local correlations are mainly found in the central portion of the Island of Montreal, where public transit is much more highly developed, especially due to the presence of the metro lines.

**Fig. 9:**
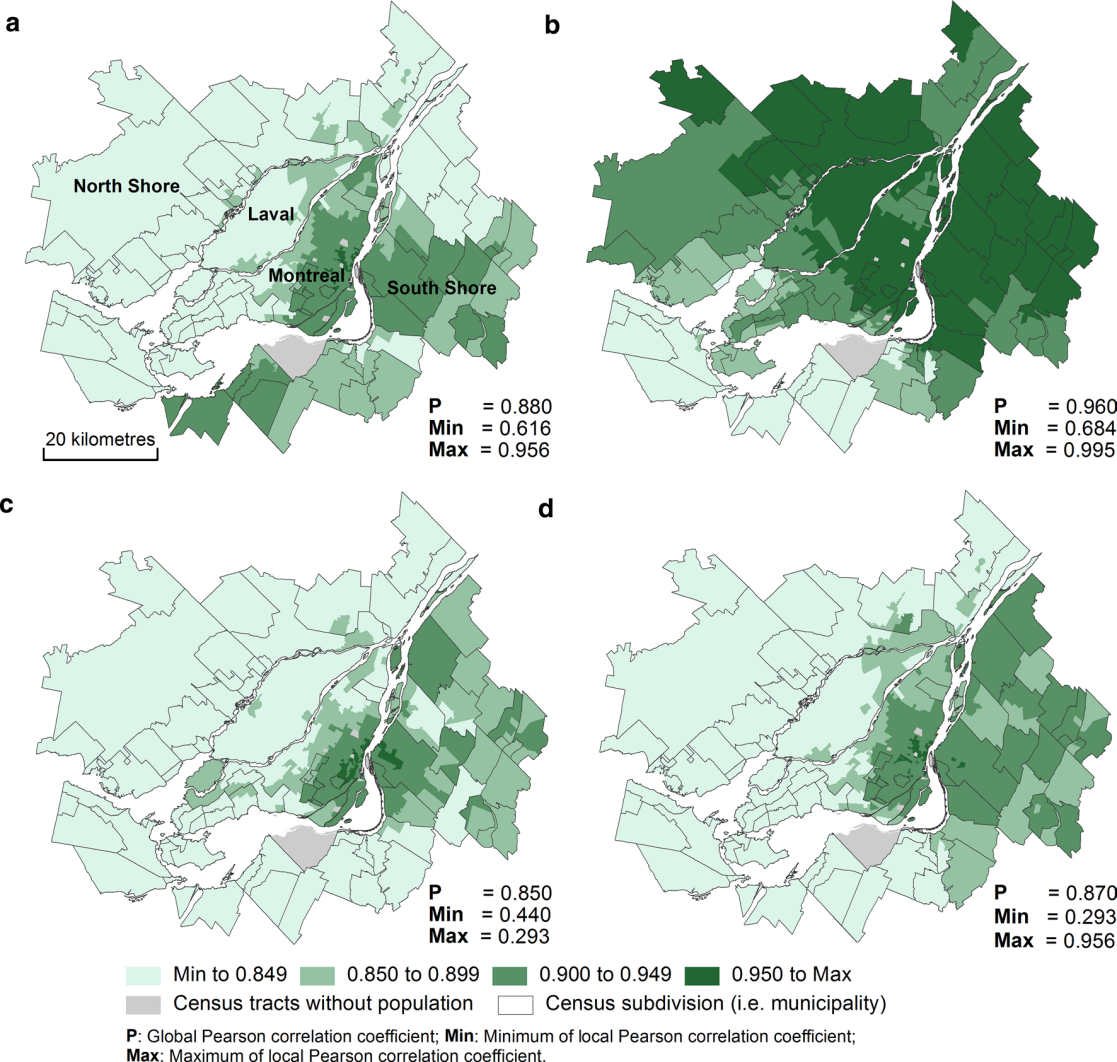
Comparing alternative types of distance between spatial units and health services using local Pearson correlations. **a** Shortest network time (on foot) versus shortest network time (by public transport). **b** Shortest network time (by car) versus shortest network time (by bicycle). **c** Shortest network time (by car) versus shortest network time (by public transport). **d** Shortest network time (by bicycle) versus shortest network time (by public transport)

Secondly, it is possible to analyze the local correlations between the four shortest network time distances (Figs. 8e, f, 9a–d). The local correlations are generally fairly strong between the shortest network time distances by car, on foot and by bicycle. On the other hand, the local correlations are much weaker with distances by public transit (Fig. 9a, c, d).

In sum, the results of the local correlations allow us to highlight two important findings. On the one hand, for a study covering the entire Montreal region, it is preferable to use network distances because Cartesian distances (especially Euclidean distances) are less accurate in suburban areas (the North and South shores). On the other hand, the distance by public transport is very different from the other types of distance (on foot, by bicycle, by car), particularly in parts of the region where the public transit system is less dense (in the eastern and western portions of the Island of Montreal and on the North and South shores).

### Aggregation errors

#### Global errors

The global analysis of aggregation errors is performed by means of Spearman’s rank correlations between the four methods of aggregation used to calculate 14 accessibility measures at the census tract level (Table 6). Due to lack of space, we only report the correlation for two types of distance (Euclidean distance and shortest network time on foot). Note that similar patterns of correlation are observed for the other types of distance.

**Table 6:** Spearman’s rank correlations between measures of the accessibility of hospitals by aggregation method

Accessibility measure	Aggregation method	Accessibility measures using Euclidean distance			Accessibility measures using shortest network time (on foot)		
		CTC^a^	WDA^b^	WBL^c^	CTC^a^	WDA^b^	WBL^c^
Minimum distance	CTC^a^	–			–		
	WDA^b^	0.994	–		0.992	–	
	WBL1^c^	0.992	0.999	–	0.988	0.997	–
	WBL2^d^	0.991	0.998	0.999	0.988	0.997	0.999
Average distance to all hospitals	CTC^a^	–			–		
	WDA^b^	0.999	–		0.999	–	
	WBL1^c^	0.999	1.000	–	0.999	1.000	–
	WBL2^d^	0.999	1.000	1.000	0.999	1.000	1.000
Average distance to three closest hospitals	CTC^a^	–			–		
	WDA^b^	0.998	–		0.997	–	
	WBL1^c^	0.998	1.000	–	0.995	0.999	–
	WBL2^d^	0.997	1.000	1.000	0.995	0.999	1.000
Average distance to five closest hospitals	CTC^a^	–			–		
	WDA^b^	0.999	–		0.998	–	
	WBL1^c^	0.998	1.000	–	0.998	1.000	–
	WBL2^d^	0.998	1.000	1.000	0.998	0.999	1.000
Number of hospitals within 500 m or 10 min	CTC^a^	–			–	0.792	0.749
	WDA^b^	0.753	–		0.792	–	0.935
	WBL1^c^	0.716	0.940	–	0.749	0.935	–
	WBL2^d^	0.704	0.923	0.985	0.740	0.921	0.962
Number of hospitals within 1000 m or 20 min	CTC^a^	–			–		
	WDA^b^	0.856	–		0.899	–	
	WBL1^c^	0.819	0.954	–	0.875	0.967	–
	WBL2^d^	0.809	0.942	0.984	0.878	0.963	0.985
Number of hospitals within 2000 m or 30 min	CTC^a^	–			–		
	WDA^b^	0.956	–		0.950	–	
	WBL1^c^	0.950	0.992	–	0.940	0.983	–
	WBL2^d^	0.949	0.991	0.998	0.935	0.982	0.994
Potential gravity model (friction parameter = 1)	CTC^a^	–			–		
	WDA^b^	0.995	–		0.996	–	
	WBL1^c^	0.996	0.999	–	0.995	0.999	–
	WBL2^d^	0.995	0.999	1.000	0.995	0.999	0.999
Potential gravity model (friction parameter = 1.5)	CTC^a^	–			–		
	WDA^b^	0.988	–		0.980	–	
	WBL1^c^	0.989	0.998	–	0.975	0.993	–
	WBL2^d^	0.988	0.997	0.998	0.981	0.995	0.996
Potential gravity model (friction parameter = 2)	CTC^a^	–			–		
	WDA^b^	0.972	–		0.952	–	
	WBL1^c^	0.979	0.990	–	0.931	0.960	–
	WBL2^d^	0.981	0.986	0.995	0.962	0.984	0.974
Two-step floating catchment area (2000 m or 30 min)	CTC^a^	–	0.977	0.976	–		
	WDA^b^	0.977	–	0.996	0.883	–	
	WBL1^c^	0.976	0.996	–	0.863	0.976	–
	WBL2^d^	0.976	0.996	0.999	0.864	0.978	0.992
Enhanced two-step floating catchment area with a slow step-decay function	CTC^a^	–			–		
	WDA^b^	0.918	–		0.908	–	
	WBL1^c^	0.908	0.986	–	0.897	0.976	–
	WBL2^d^	0.908	0.986	0.997	0.889	0.972	0.991
Enhanced two-step floating catchment area with a fast step-decay function	CTC^a^	–			–		
	WDA^b^	0.916	–		0.905	–	
	WBL1^c^	0.906	0.986	–	0.894	0.975	–
	WBL2^d^	0.907	0.986	0.997	0.887	0.971	0.991
Enhanced two-step floating catchment area with a gradient function	CTC^a^	–			–		
	WDA^b^	0.917	–		0.907	–	
	WBL1^c^	0.908	0.987	–	0.895	0.975	–
	WBL2^d^	0.909	0.987	0.997	0.887	0.972	0.991

^a^Aggregation method based on census tract centroid (the least accurate method)

^b^Aggregation method based on the population-weighted mean of the accessibility measure for dissemination areas within census tracts

^c^Aggregation method based on the population-weighted mean of the accessibility measure for blocks within census tracts

^d^Aggregation method based on the population-weighted mean of the accessibility measure for block centroids (adjusted with the land use map) within census tracts (the most accurate method)

Correlations between the four aggregation methods are high (>0.9) for all accessibility measures except for the number of hospitals within 500 and 1000 m or within 10 and 20 min. For example, correlation between the least and most accurate aggregation methods (CTC and WBL2) is 0.704 for the number of hospitals within 500 m and 0.740 for those within 10 min on foot. This means that if we want to assess service provision in a close-proximity area around a census tract, it is preferable to use an aggregation method that precisely accounts for the distribution of population within it; if not, the risk of error may be considerable.

#### Local errors

A second stage of comparison of aggregation methods consists in assessing the absolute difference between the geographical accessibility results obtained with the CTC and WBL2 aggregation methods. The descriptive statistics for local errors are reported in Table 7 for hospitals. Not surprisingly, the local errors are on the whole quite small, though not insignificant: for example, compared with the most accurate method, the census tract centroid method misestimates the distance to the closest hospital by an average of 236 m (Euclidean distance) and 4.17 min (on foot). Up to the third quartile (75%), the local errors are still quite small: for 75% of census tracts, the error associated with the census tract centroid approach is <218 m or 3.65 min. However, in 10% of cases, the error is >649 m and 10.22 min, and in 5% of census tracts the error is >1.1 km and 17 min (Table 7). Despite the high correlations, significant errors in the measurement of geographical accessibility can occur in a small number of cases.

**Table 7:** Aggregation errors in measures of the accessibility of hospitals at the census tract level

Absolute difference between accessibility measure obtained from CTC^a^ and WBL2^b^ aggregation methods	Mean	Percentiles (%)						
		5	10	25	50	75	90	95
Euclidean distance (m)								
Minimum time distance	236.35	5.24	11.17	33.29	88.44	218.39	649.26	1104.88
Average distance to all services	202.62	2.05	4.01	13.41	42.99	146.13	617.01	1022.88
Average distance to three closest services	189.97	3.01	6.15	16.40	47.73	159.28	527.03	963.20
Average distance to five closest services	184.45	3.05	5.42	15.31	46.69	140.41	530.94	997.41
Number of services within 500 m	0.05	0.00	0.00	0.00	0.00	0.00	0.17	0.43
Number of services within 1000 m	0.10	0.00	0.00	0.00	0.00	0.04	0.37	0.59
Number of services within 2000 m	0.17	0.00	0.00	0.00	0.00	0.25	0.56	0.82
Potential gravity model (friction parameter = 1)	0.00	0.00	0.00	0.00	0.00	0.00	0.00	0.00
Potential gravity model (friction parameter = 1.5)	0.00	0.00	0.00	0.00	0.00	0.00	0.00	0.00
Potential gravity model (friction parameter = 2)	0.00	0.00	0.00	0.00	0.00	0.00	0.00	0.00
2SFCA (2000 m or 30 min)	0.22	0.00	0.00	0.01	0.06	0.23	0.57	1.09
E2SFCA with a slow step-decay function	1.93	0.00	0.00	0.00	0.02	1.47	5.73	9.53
E2SFCA with a fast step-decay function^a^	4.07	0.00	0.00	0.00	0.19	2.54	10.05	16.20
E2SFCA with a gradient function	1.97	0.00	0.00	0.00	0.08	1.41	5.91	9.73
Shortest network time (on foot) (min)								
Minimum time distance	4.17	0.10	0.25	0.67	1.61	3.65	10.22	17.17
Average distance to all services	3.49	0.05	0.12	0.34	1.00	2.72	9.37	16.48
Average distance to three closest services	3.55	0.07	0.15	0.40	1.18	3.05	8.97	15.62
Average distance to five closest services	3.44	0.07	0.14	0.36	1.05	2.86	8.30	15.48
Number of services within 10 min	0.06	0.00	0.00	0.00	0.00	0.00	0.24	0.45
Number of services within 20 min	0.12	0.00	0.00	0.00	0.00	0.11	0.46	0.68
Number of services within 30 min	0.17	0.00	0.00	0.00	0.00	0.22	0.58	0.83
Potential gravity model (friction parameter = 1)	0.00	0.00	0.00	0.00	0.00	0.00	0.00	0.00
Potential gravity model (friction parameter = 1.5)	0.00	0.00	0.00	0.00	0.00	0.00	0.00	0.00
Potential gravity model (friction parameter = 2)	0.00	0.00	0.00	0.00	0.00	0.00	0.00	0.00
2SFCA (2000 m or 30 min)	0.92	0.00	0.00	0.00	0.00	0.56	2.59	4.83
E2SFCA with a slow step-decay function	1.70	0.00	0.00	0.00	0.01	1.06	3.95	7.67
E2SFCA with a fast step-decay function^a^	3.29	0.00	0.00	0.00	0.04	1.85	7.33	13.20
E2SFCA with a gradient function	2.27	0.00	0.00	0.00	0.01	1.22	4.71	10.38

^a^Aggregation method based on census tract centroid (the least accurate method)

^b^Aggregation method based on the population-weighted mean of the accessibility measure for block centroids (adjusted with the land use map) within census tracts (the most accurate method)

**Fig. 10:**
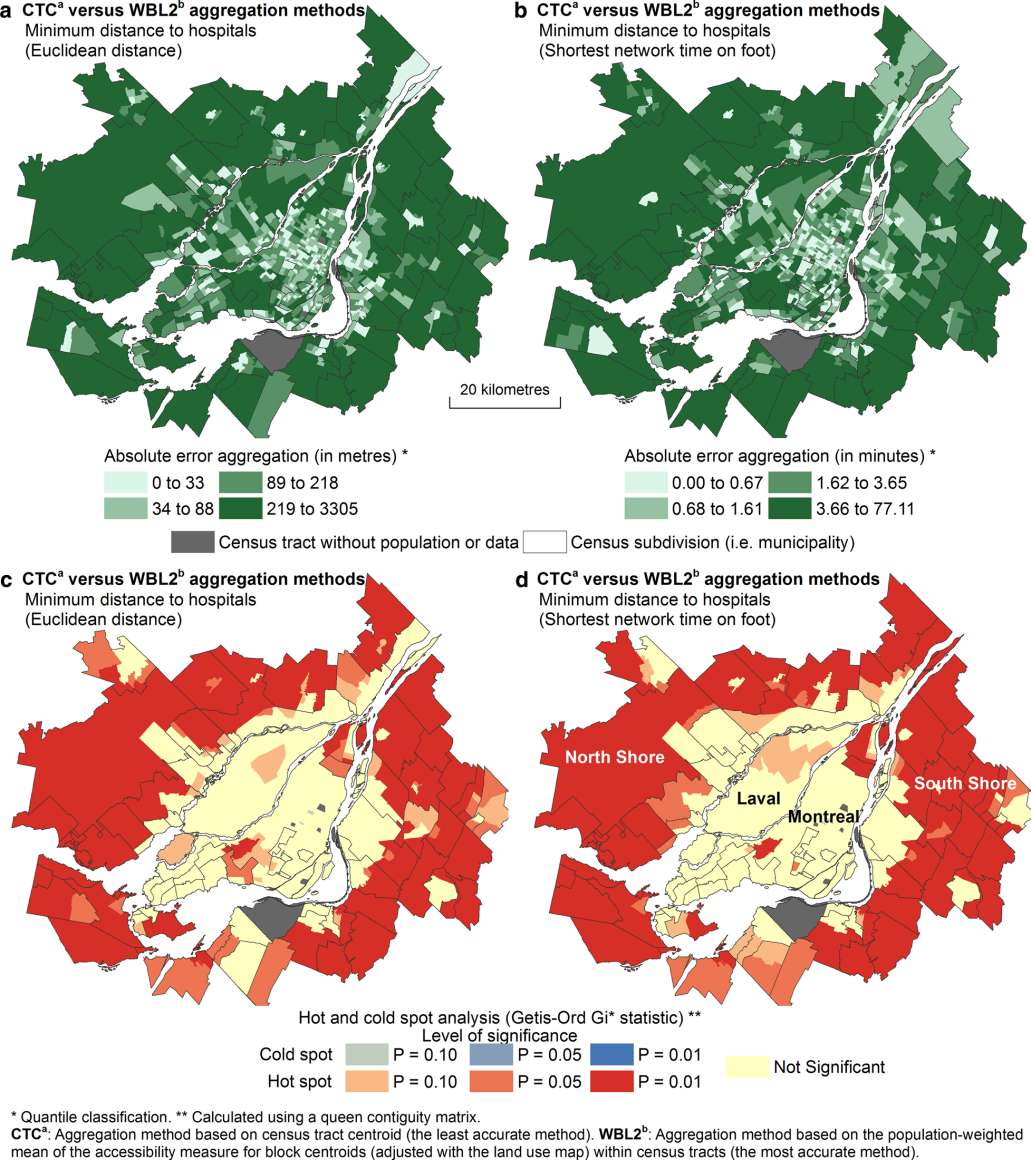
Evaluating local aggregation errors

Absolute differences between aggregation methods for the closest hospital computed using Euclidean distance and shortest network time (on foot) are further mapped in Fig. 10a, b. Again, stronger absolute aggregation errors are observed in suburban areas on the South and North shores of the CMA; errors remain smaller in central areas of the Island of Montreal. Moreover, the use of the local Getis–Ord Gi* statistic clearly shows that hot spots in aggregation errors are located on the North and South shores (Fig. 10c, d). This shows that, in suburban areas, where the surface area of census tracts is greater than in central neighbourhoods, it is preferable to use an accurate aggregation method to prevent significant measurement errors.

### Sensitivity analysis (SA) and uncertainty analysis (UA)

The sensitivity analysis was performed by using three transformations (z-score standardization, normalization on a scale of 0–1, and use of ranks). Table 8 reveals several interesting findings. First, the uncertainty factor generating the most variance is the accessibility measure, with 74–86% of the total variance, depending on the transformation used (first order sensitivity index). When placed in interaction with the type of distance, the accessibility measures also explain 10–20% of the total variance (second order sensitivity index) for a total sensitivity index of over 90.

**Table 8:** Results of sensitivity analysis (Sobol’s indexes)

Transformation	Z-score	0–1	Ranks
First order sensitivity index			
Distance type (n = 6)	3.34	6.05	3.12
Aggregation method (n = 4)	0.01	0.10	0.83
Accessibility measure (n = 14)	86.00	79.91	74.17
Second order sensitivity index (interaction)			
Distance type versus aggregation method	0.02	0.01	0.24
Distance type versus accessibility measure	10.19	13.52	19.76
Aggregation method versus accessibility measure	0.15	0.31	1.33
Total explained variance	99.71	99.89	99.44
Total sensitivity index			
Distance type	13.55	19.58	23.12
Aggregation method	0.18	0.41	2.40
Accessibility measure	96.34	93.73	95.25

The second most important uncertainty factor is the type of distance, with 3–6% of the total variance (first order sensitivity index); 10–20% of the variance when placed in interaction with the accessibility measure (second order sensitivity index); and a value for the total sensitivity index of 13.55–23.12. Thirdly, the impact of the aggregation method is much more limited: <1% for the first order sensitivity index; and a value for the total sensitivity index of between 0.18 and 2.40.

**Fig. 11:**
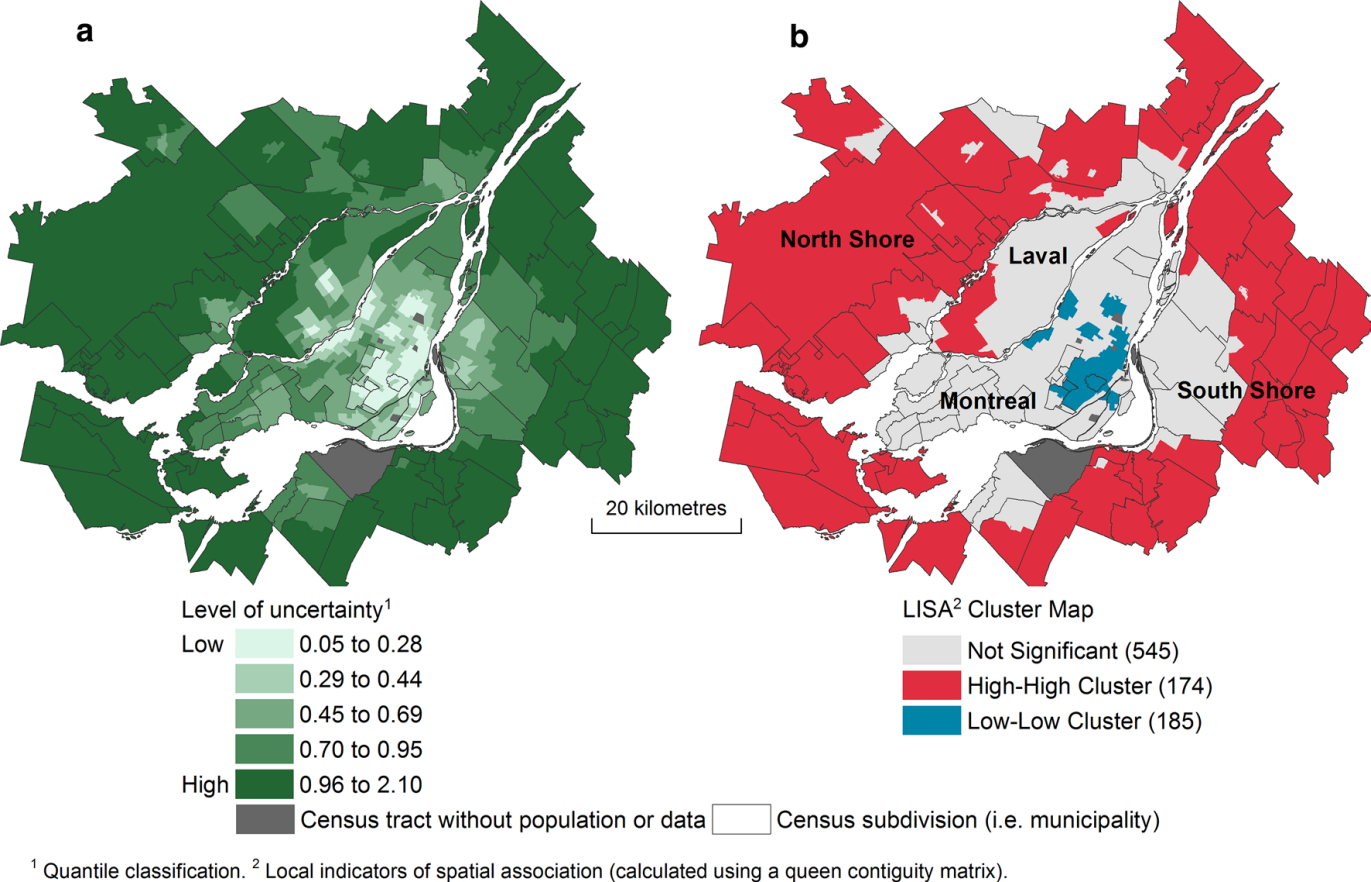
Results of uncertainty analysis. **a** Spatial distribution of uncertainty. **b** Spatial clusters of uncertainty

The UA results, mapped in Fig. 11a, b, clearly indicate areas of strong uncertainty on the North and South shores, whereas central neighbourhoods show lower levels of uncertainty. In other words, the choices made regarding the three parameters—distance types, accessibility measures and aggregation methods—have relatively little impact in the development of the assessment of potential geographic access in central neighbourhoods, unlike the case on the North and South shores.

## Discussion

The SA results allowed us to show that the evaluation of potential geographic access may vary a great deal depending on the accessibility measure and, to a lesser degree, the type of distance and aggregation method. This is not surprising, as the 14 accessibility measures selected refer to conceptualizations of potential geographic access that are very different from one another. The choice of this parameter should thus be given considerable attention and have a specific justification as it helps to make the results vary substantially. It is then relevant to calculate several measures that enable potential geographic access to be described in all its complexity. For example, measures based on both supply and demand dimensions (2SFCA and gravity models) are better adapted to general, large-size services (e.g. hospitals), whereas measures of immediate proximity or cumulative opportunities are more suited to describing less common, specialized services (specialized centres). Moreover, these cumulative opportunity measures are especially well adapted to describing the supply of services within an immediate environment (within one mile, for example). That is why they are often used in health studies on food deserts [e.g. 28, 69, 70] or the food environments around schools [e.g. 71–73]. It should also be remembered that these accessibility measures produce variables that may be either continuous or discrete, which may result in a particular one being chosen in keeping with the study design.

Although the choice of the type of distance has less impact on the results obtained, it clearly interacts to a certain extent with the measure of accessibility. The interactions between these two sources of uncertainty generate more variation than the method of aggregation alone. For example, in 2SFCA methods, the catchment areas may be either larger or smaller depending on the type of distance chosen (car travelers can go further than pedestrians), and the same goes for measures that count the number of services within a specific radius. So this choice also requires careful consideration, in taking into account possible interactions with the latter measure.

Moreover, although Cartesian distances (Euclidean and Manhattan) are strongly correlated with the four network distances, local variations are nonetheless observed, notably in suburban areas. Given that it has become much easier to calculate network distances—because of free access to geographical data and highly effective tools (GIS or online services)—the use of Cartesian distances in urban areas is no longer preferable today. Indeed, the time required for the computation of numerous network distances is no longer a limitation.

Another aspect should be mentioned concerning the comparison of the types of distance. The correlations have been shown to be weaker between public transit and other network distances. This can be explained by the unequal distribution of public transport (especially the subway) across the study area. The same remark also applies, although to a lesser extent, to cycling infrastructure. We also found strong correlations between network distances on foot, by bicycle and by car, which might lead one to believe that using one or the other of these types of distance comes down to applying a simple multiplying factor, which is not the case. The impact of topography on pedestrians’ or cyclists’ speeds is much greater than for car drivers. In a city with a more pronounced topography (e.g. San Francisco, La Paz), these correlations would certainly have been weaker. Finally, the correlations with Cartesian distances were also weaker for peripheral areas than for central areas. In the Montreal context, this is in part explained by the presence of the bridges that link the Island to the North and South shores. In comparison, if we had conducted the study in the New York City area, the local correlations between Cartesian versus network types of distance would probably have been very high for census tracts on the island of Manhattan, and weaker for those located in Brooklyn, Queens, Staten Island and Jersey City because of the bridges.

Finally, although the influence of the aggregation method is fairly marginal globally, we have nonetheless shown that errors in accuracy caused by the lack of an aggregation method can be important locally, especially in suburban areas, where census tracts mostly have lower population densities and where land use is largely non-residential. Because the accessibility of health services may be more problematic in suburban areas than in more central urban areas, geographical accessibility studies should be based on the most accurate aggregation method. The question of the use of an aggregation method is especially important when accessibility measures calculated on the level of census tracts are introduced as dependent variables into models for predicting health outcomes. Consider the classic example of a multilevel model with individual variables (level 1), socioeconomic variables and measures of the accessibility of health services or health-related resources at the census tract level (level 2). If the accessibility measures are not calculated by using an aggregation method—in other words, if they are obtained by only using the census tract centroids—that could lead to errors or lack of precision in the estimation of the impact of the accessibility of health services or health-related resources on health.

## Conclusion

This article evaluates the potential geographic access to urban health services using 14 accessibility measures, six types of distance and four aggregation methods. Based on these three parameters, 336 indicators of geographic access at the census tract level have been obtained. A sensitivity analysis has shown that the parameters that create the greatest variation in the evaluation of potential geographic access are, in descending order: the accessibility measures and, to a far lesser extent, the type of distance and the aggregation method used. An uncertainty analysis also made it possible to show that inaccuracies in the evaluation of geographic access are much greater in the suburbs than in central neighbourhoods.

In sum, in order to accurately assess potential geographic access to health services in urban areas, it is particularly important to choose a precise type of distance and aggregation method so as to limit inaccuracies in measurements. Then, depending on the research question and/or research objectives, the choices of the type of network distance (according to the mode of transportation) and of a number of accessibility measures should be carefully considered and adequately justified.

## Abbreviations

2SFCA: two-step floating catchment area
E2SFCA: enhanced two-step floating catchment area
3SFCA: three-step floating catchment area
CMA: census metropolitan area
CTC: census tract centroid
GIS: geographic information systems
GTFS: general transit feed specification
SA: sensitivity analysis
UA: uncertainty analysis
WBL1: population-weighted mean of the accessibility measure for blocks within census tracts (WBL1)
WBL2: population-weighted mean of the accessibility measure for blocks (adjusted with the land use map) within census tracts
WDAC: population-weighted mean of the accessibility measure for dissemination areas within census tracts

## Footnotes

1
http://www.betterbybicycle.com/2014/09/how-accurate-are-google-maps-cycling.html.

2
http://transit.melindamorang.com/.
